# Structural validation of ORTO-11-ES for the diagnosis of orthorexia nervosa, Spanish version

**DOI:** 10.1007/s40519-018-0573-3

**Published:** 2018-09-08

**Authors:** María Laura Parra-Fernandez, Teresa Rodríguez-Cano, Maria José Perez-Haro, María Dolores Onieva-Zafra, Elia Fernandez-Martinez, Blanca Notario-Pacheco

**Affiliations:** 1 Faculty of Nursing, University of Castilla-La-Mancha, Ciudad Real, Spain; 2 Head of Mental Health, Castilla la Mancha Health Services, Toledo, Spain; 3 Biostatech Advice, Training and Innovation in Biostatistics, S.L Santiago de Compostela, A Coruña, Spain; 4 Faculty of Nursing, University of Castilla-La-Mancha, Cuenca, Spain

**Keywords:** Orthorexia nervosa, ORTO-11-ES, University students, Confirmatory factor analysis

## Abstract

**Purpose:**

The ORTO-11-ES questionnaire is a tool to assess the pathological obsession displayed by some individuals regarding healthy eating. The aims of this study were (1) to confirm the factor structure of the Spanish version of ORTO-11-ES using confirmatory factor analysis (CFA) and (2) to examine the possible association between the ORTO-11-ES score, gender and body mass index (BMI).

**Methods:**

The sample comprised 492 students from the University of Castilla la Mancha, Spain. Of these, 280 were women (56.9%). Participants were surveyed using the ORTO-11-ES questionnaire.

**Results:**

The confirmatory factor analysis (CFA) supported the 11 elements and 3 domains of this tool as the better fitting model; for the Comparative Fit Index (CFI) and the Tucker–Lewis Index (TLI), the values were 0.94 and 0.91, respectively, and the Root Mean-Square Error of Approximation (RMSEA) was 0.058. The tendency towards orthorexic behavior is more associated with the female gender. The BMI had no influence on the tendency for ON.

**Conclusions:**

This study is the first attempt to confirm the three-factor structure of a Spanish version of the ORTO-15 questionnaire. These findings suggest that the ORTO-11-ES may be a valuable tool for identifying subjects with specific eating behavior patterns. This information may be useful for health professionals involved in the research, development and implementation of interventions catered to individuals suffering from this eating disorder.

**Level of evidence:**

Level V, descriptive cross-sectional study.

**Electronic supplementary material:**

The online version of this article (10.1007/s40519-018-0573-3) contains supplementary material, which is available to authorized users.

## Introduction

Orthorexia nervosa (ON) is the term given to a constellation of pathological attitudes and behaviors related to attempts to eat only “healthy” or “pure” food [1]. When extreme, ON can lead to physical, psychological, and social impairments, such as malnutrition, isolation, and decreased quality of life [1, 2].

The potential severity of the physical and psychological effects of ON vary considerably, depending on the duration of the behaviors, the specifics of an individual’s eating choices, the level of adherence to food rules, and any underlying or co-occurring conditions. Previously reported physical effects include protein-energy malnutrition, iron-deficiency anemia, vitamin excesses and deficiencies, osteoporosis, cardiovascular abnormalities, hypotension, and trace-element deficiencies [2–5]. From a psychological point of view, although empirical long-term studies are lacking, there is anecdotal evidence that this type of dietary extremism is associated with a tendency for affected individuals to seek isolation, limiting social interaction with others [1, 6]. If unable to conform to food rules or dietary ideals, an individual with ON tendencies may develop feelings of self-blame, distorted cognitions, and even psychosis. Alterations in brain chemistry, specifically dopamine and serotonin, may cause feelings of euphoria and/or anxiety [7, 8].

In recent years, ON has become a popular subject within the scientific community, with the publication of numerous studies evaluating the psychometric properties of the available questionnaires used for the assessment of this disorder [9–13]. Epidemiological research on ON has been conducted across various demographic groups and in different countries [14–19]. Incidence rates vary greatly, from 1 to 87%, suggesting either high variability of ON between groups or low reliability of the survey instruments used.

The most widely used questionnaire to date for the detection of orthorexic behavior is the ORTO-15, developed by Donini et al., which includes 15 multiple choice items [9]. This questionnaire combines the informal orthorexia self-test devised by Bratman with phobic and obsessive personality traits as described by the Minnesota Multiphasic Personality Inventory (MMPI) [20, 21]. The original authors of this tool assumed three factors: the cognitive-rational factor (items 1,5,6,11,12, 14), the clinical factor (items 3, 7, 8, 9, 15) and the emotional factor (items 2, 4, 10, 13) which evaluate three areas of decline related with ON. In the literature, acceptable confidence levels have been demonstrated for the ORTHO-15 in other countries [9–13]. Table 1 displays the psychometric properties of the different validated versions [10–13, 22].

**Table 1:** Summary of study characteristics and best fit models reported in previous ORTO-15 CFA studies

References	Language	Name of measure	Adaptation (items discarded)	Validity reported (α Cronbach)	Method: CFA/EFA	Factor structure	Fit indices
Arusoğlu et al. [10]	Turkish	ORTO-11 Turkish	1, 2, 9 and 15	0.62	CFA	One factor	
Varga et al. [11]	Hungarian	ORTO-11-HU	5, 6, 8 and 14	0.82	CFA	One factor	*X* ^2^ = 230.8; CFI = 0.92; TLI = 0.90; RMSE = 0.076
Brytek et al. [12]	Polish	ORTO-9	1, 2, 8, 9,13 and 15	0.64	EFA, CFA	Two factors	*X* ^2^ = 35.697; CFI = 0.953 ; RMSE = 0.053
Missbach et al. [13]	German	ORTO-9-GE	1, 2, 8, 9, 13 and 14	0.67	CFA	One factor	*X* ^2^ = 83.865; CFI = 0.947; TLI = 0.92 ; RMSE = 0.048

*CFA* confirmatory factor analysis, *EFA* exploratory factor analysis, *CFI* comparative fit index, *TLI* Tucker–Lewis Index, *RMSEA* root-square error or approximation

The Spanish version (ORTO-11-ES) [22] has demonstrated to have good reliability and good factorial validity. The authors suggest a three-factor solution that explains the 32.44, 11.45 and 9.15% of the total variance, respectively. The final version is a test featuring 11 items (1, 2, 3, 4, 6, 7, 9, 10, 11, 12 and 13) from the original ORTO-15 test, having removed four elements (5, 8, 14 and 15). The internal consistency of this abbreviated 11-item version (ORTO-11-ES) is acceptable with an alpha Cronbach value of 0.80 [22]. The structural comparison with other validations made in different languages shows that in the Turkish, Hungarian and German versions [11–13] a single structure factor is reported. Only the Spanish version [22] has been able to replicate the three-factor structure as reported in the original Italian version [23].

Considering that the factorial validation of the original version identified a three-dimensional structure [23], this study attempted to test this hypothesis and, therefore, the primary aim of the present study was to confirm the three-factor structure of the Spanish version ORTO-11-ES [22], based on confirmatory factorial analysis (CFA) in a sample of university students from the University of Castilla La Mancha, Spain. The construct validity of the Spanish version of the ORTO-11-ES should be confirmed as this would provide further scientific consistency to the validation of this questionnaire. The growing interest on the topic of ON in research contexts, both in terms of its characteristics and epidemiology, suggests the need for further studies to explore the psychometric validity of these instruments. To date, due to the variability of prevalence rates reported using this instrument in different countries, authors such as Missbach et al. have questioned the limitations of the ORTHO-15 [24]. Some authors suggest that it may only identify healthy eating and not a pathological obsession with healthy eating [18]. To the best of our knowledge, no prevalence rates have been published in the Spanish population based on the ORTO-11-ES instrument; therefore, more studies are needed based on the Spanish population to further inform the debate regarding the use of this instrument.

The secondary aim of this study was to assess two demographic variables which may be related to eating disorders: gender and body mass index (BMI) [9, 13, 25–27]. To our knowledge, no study has been performed to date in Spain researching this possible relationship in a Spanish population.

The findings from this study will help the scientific community devise appropriate tools for the detection of this problem and will shed light on the possible factors related to the development of ON.

## Methods

The study protocol was approved by the Ethics Committee of the Castilla-La-Mancha University Hospital, in compliance with the ethical standards established in the 2008 Helsinki Declaration. The participants were recruited from the Ciudad Real campus within the University of Castilla La Mancha (Spain) and studied a bachelor’s degree in health sciences or a bachelor’s degree in engineering and architecture. Informed consent was obtained from all individual participants included in the study. Participants had to be enrolled in the University in the 2017/18 year to satisfy the inclusion criteria. There were no exclusion criteria. The data were gathered via the Internet using the JotForm platform and the students participated voluntarily in the study. Students were requested to complete an online survey developed by the authors.

### Instruments

#### Demographic questionnaire

The students indicated their age, gender, degree, and current height and weight.

#### ORTO-11-ES questionnaire

The ORTO-11-ES questionnaire is a self-report measure consisting of 11 items with multiple choice responses and based on a Likert scale (always, often, sometimes, never) to measure three underlying factors related to eating behavior: cognitive rational (1, 4, 5 and 11), clinical (2, 3 and 6) and emotional aspects (7, 8, 9 and 10) [22]. The ORTO-11-ES was administered in Spanish and was completed in approximately 10 min. The lower the score, the greater the indication of the behavior or attitudes related to orthorexia. A previous validation study using the ORTO-11-ES concluded that a cut-off point of < 25 (efficiency 84%, sensitivity 75% and specificity 84%) was considered to be the most appropriate cut-off point for suggesting the presence of ON tendencies [22].

### Statistical analysis

Confirmatory factor analysis was used to assess the internal structure of the Spanish version of the questionnaire on orthorexia nervosa. From the onset, two types of models were proposed: the first model was based on the three-factor structure found by Parra-Fernández et al. [22], which resulted from applying the principal component analysis (PCA). Thereafter, there has been an attempt to simplify this model to a single dimension, as proposed in other versions of the same questionnaire in different languages [10, 11, 13]. We used the WLSMV estimator, designed for use in small- and medium-sized samples [28]. The model adjustments were evaluated using the Chi-square goodness-of-fit test, where zero indicates perfect fit. Three model fit indicators were calculated. For the Steiger–Lind root mean square error of approximation (RMSEA), < 0.08 is indicative of acceptable model fit and < 0.05 indicates a correct model fit. For the Tucker–Lewis index (TLI) and the Bentler comparative fit index (CFI), > 0.90 means acceptable fit and > 0.95 indicates appropriate fit [29]. The internal consistency reliability for each factor was evaluated using the alpha Cronbach coefficients.

The text below examines the differences between the mean scores for the ORTO-11-ES according to the gender and BMI of participants. The normality of the continuous variables is examined via the Shapiro–Wilk test. In the case of normally distributed variables, parametric methods were used (*t* test, ANOVA, Pearson), whereas, in the case of non-normality in the study variables, non-parametric methods were used such as the Mann–Whitney, Kruskal–Wallis, and Spearman tests.

These analyses were performed using the R statistical software [30]. Additionally, the ‘lavaan’ [31] package was used to calculate the fit indexes.

## Results

Our study sample consisted of 492 students, 280 of whom self-identified as female (56.9%), with mean ages of 20.35 years (± 3.37) for women and 19.46 (± 2.44) years for men. The mean body mass index (BMI), calculated based on the weights and heights reported by participants was 22.21 (± 7.97) for women and 23.43 (± 3.81) for men.

### Confirmatory factor analysis (CFA)

The CFA of the ORTO-11-ES questionnaire was used to test and compare between two hypothetical models (Table 2).

**Table 2:** Summary of the proposed models

Type of model	Dimensions	Items
Model I	Rational	1, 4, 5 and 11
	Behavioral	2, 3 and 6
	Emotional	7, 8, 9 and 10
Model II	Orthorexia nervosa	1, 2, 3, 4, 5, 6, 7, 8, 9, 10 and 11

### Model I

**Fig. 1:**
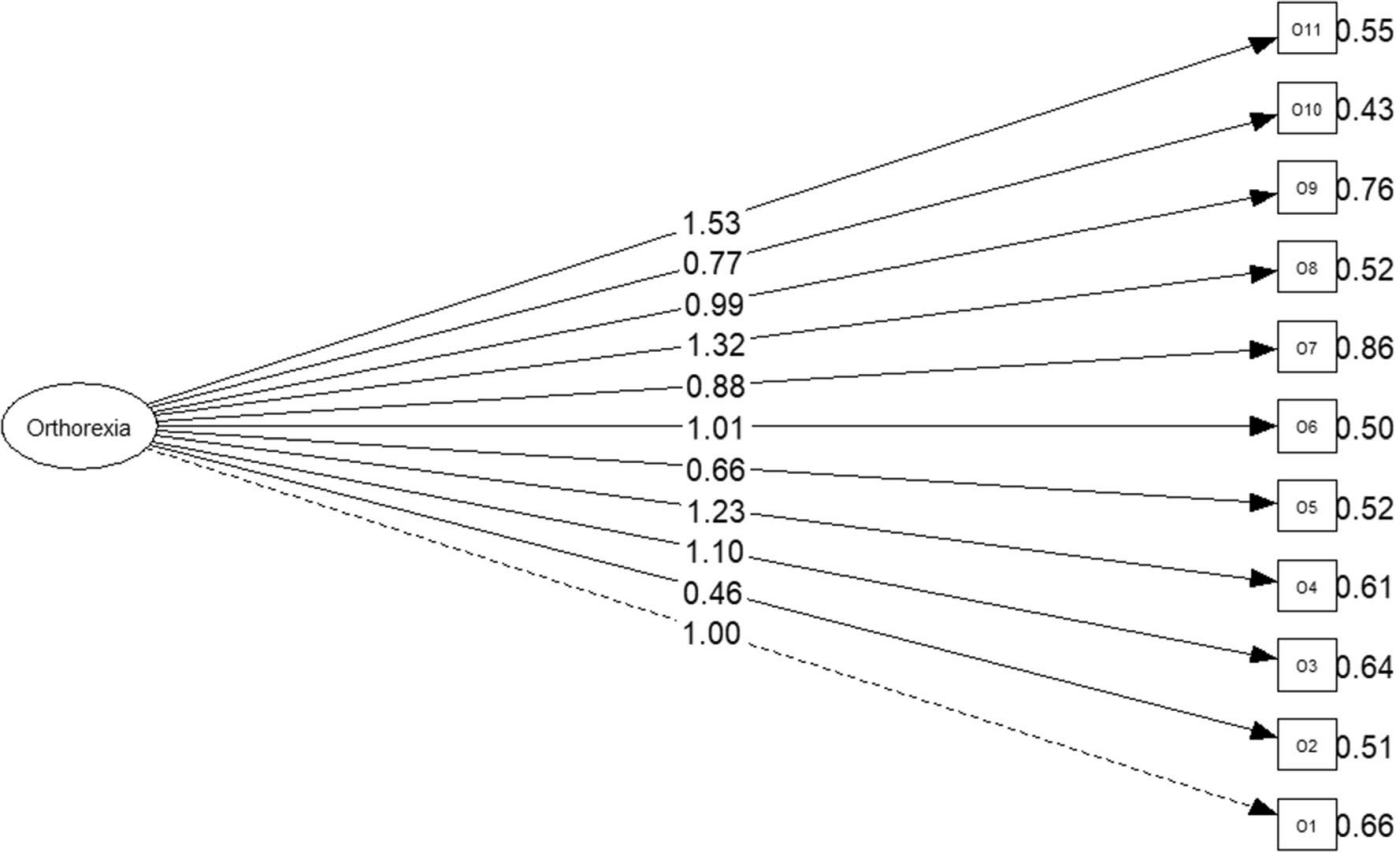
ORTO-11 ES (1-factor structure)

The CFA confirmed that the three-dimensional solution has an excellent goodness-of-fit (*χ*^2^ = 64.13, *p* = 0.01; CMIN/DF = 1.17; CFI = 0.99; TLI = 0.98; RMSEA = 0.03, PCLOSE = 0.9; SRMR = 0.04; see Fig. 1). Error terms were not added, as, according to the literature, these must only be added in the event that the modification indexes are equal or above 40 and in our case, all are < 40. The internal consistency of each of these dimensions was 0.63, 0.57 and 0.62.

### Model II

**Fig. 2:**
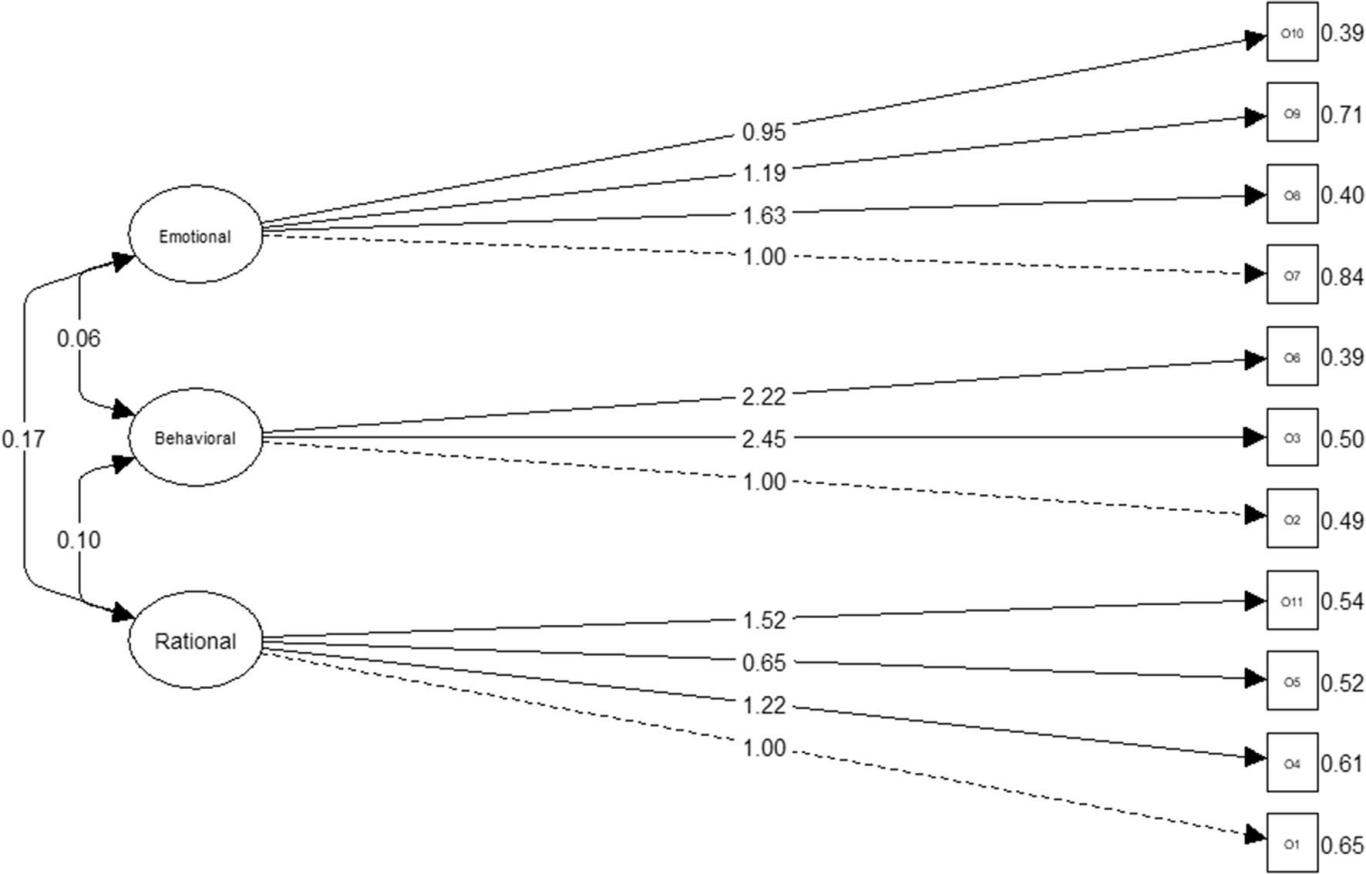
ORTO-11 ES (3-factor structure)

The confirmatory factorial analysis of a single factor presents a good goodness-of-fit (*χ*^2^ = 101.03; *p* = 0.00; CMIN/DF = 2.30; CFI = 0.97; TLI = 0.96; RMSEA = 0.05, PCLOSE = 0.41; SRMR = 0.06; see Fig. 2). The internal consistency of the 11 items presents a Cronbach alpha of 0.79. The goodness-of-fit indexes for both models are presented in Table 3.

**Table 3:** Comparison of goodness-of-fit indexes among the unidimensional and three-dimensional models of the ORTO-11-ES questionnaire

	Fit indices							
Model	${\chi ^2}$	*p* value	CMIN/DF	CFI	TLI	RMSEA	PCLOSE	SRMR
Model I	64.13	0.01	1.17	0.99	0.98	0.03	0.96	0.04
Model II	101.03	0.00	2.30	0.97	0.96	0.05	0.41	0.06

*CFI* index comparative fit index, *TLI* Tucker–Lewis Index, *RMSEA* root mean square error of approximation, *SRMR* standardized root mean square residual, *Model I* tree-factor model of Parra et al., *Model II* one-factor model

### Relationships between ORTO-11-ES score, gender, and BMI

ORTO-11-ES scores can range from a minimum of 12 and a maximum of 44, with the lowest scores indicating the presence of the most orthorexic behaviors. The mean score obtained by the participants of the ORTO-11-ES questionnaire was 28.44 (SD = 5.67). Individual ORTO-11-ES scores followed a normal distribution. The mean score obtained by male participants (30.02 ± 5.17) significantly differed from the mean score obtained by women (27.25 ± 5.74); *t* = 5.6212, *p* = 0.00. This implies that women obtained significantly lower scores than men. The BMI values (calculated using self-reported height and weight) were interpreted based on the BMI classification published by the World Health Organization, where a BMI of below 18.5 was categorized as underweight, 18.5–24.99 as normal, 25.0–29.99 as overweight (pre-obese), 30 and above as obese [32]. The correlation between the total score and the BMI is very weak and negative (*ρ* = − 0.13, *p* = 0.003). Furthermore, regarding the differences between the scores obtained according to the BMI categorization, our results prove that significant differences do not exist among the total scores obtained according to the BMI (*F* = 1.83, *p* = 0.14).

## Discussion

This study used self-report methods to examine the ON tendency of a student population measured using the ORTO-11-ES questionnaire [22]. The study aims were to confirm the factor structure found in the ORTO-11-ES and to perform an analysis of the possible relationship between the ORTO-11-ES score and BMI and gender present in individuals with lower ORTO-11 scores.

For this first study aim, we performed a CFA to confirm the three-dimensional factorial structure of the ORTO-11-ES questionnaire [22]. Interestingly, in contrast to prior studies [10–13] our analysis demonstrated that the three-factor structure adjusts significantly better than a single factor model. As opposed to the exploratory factorial analysis, the CFA has an essential advantage, as it provides explicit estimations of the parameters of error variance and, therefore, it is possible to evaluate and correct the measurement error. In this sense, the Spanish version is the only one that proposes the three-structure model originally designed by Donini et al. [9]. It is worth noting that our Cronbach alpha value reported for each of the dimensions does not suggest the use of each dimension separately, but rather as part of the total scale. However, the composition of each of the dimensions of the Spanish version does not coincide with the original proposal. The fact that items from the different areas covered by the questionnaire have suffered modifications leads us to a more in-depth debate regarding the construction and/or meaning of these items, from a purely observational point of view. Indeed, the suitability of some of the items has been debated by some researchers, for example, Missbach et al. [13] who question whether item 1 regarding calories provides valid information, considering that, generally speaking, a person with orthorexia is considered to worry more about the quality of food as opposed to the quantity of the same. Likewise, the idea that orthorexia may be a subphase within the healing process of other eating disorders such as anorexia, as other research has suggested [33, 34], may favor the inclusion of this item. The many divergent study results and clinical opinions about ON suggest that the scientific and medical communities must continue to work together toward a clearer understanding of the disorder and its symptoms, as well as for the development of a more reliable standard screening tool.

Another aspect of the questionnaire worth considering is temporality, which the individual must consider when providing responses. For example, item 3 refers to behavior in the last 3 months. However, this makes it impossible to evaluate whether the subject has recently had a health problem, and thus needs to exercise greater caution temporarily by following a strict diet, i.e., by not including certain food items or by exercising certain restrictions. This could lead to a bias in the responses provided to certain items and is, therefore, an element that warrants consideration [35].

The findings of the present study reveal that individuals who have scored positively for the ORTO-11-ES did not differ significantly with regard to their BMI (*p* ≥ 0.05). These results confirm some previous studies [11, 36–38]. However, others report a positive relationship between a higher BMI and a greater tendency for all the components of ON [17, 25, 37]. Although the results of the different studies are contradictory, this variable should not be overlooked. Indeed, it should be considered when developing new tools for the detection of ON-based research studies seeking to provide more knowledge on the same and, therefore, to reassess whether or not this variable may be predictive of an orthorexic behavior. Furthermore, according to the most recent proposed diagnostic criteria published by Dunn and Bratman [39], malnutrition and weight loss are the new diagnostic criteria proposed for ON. Moroze et al., however, suggest that a positive relationship does not have to exist between an individual’s weight loss and a greater tendency to ON. This is because individuals with normal bodyweight can also suffer from this disorder. The primary care teams are those who, via a comprehensive clinical interview, can determine and detect those patients who acknowledge tendencies for obsessive worrying regarding healthy diets despite being at their ideal weight [2].

Our findings revealed that the female participants displayed a greater tendency for orthorexia according to the ORTO-11-ES survey compared with men. These findings support some prior reports [40, 41]. The study by Dell’Osso included a sample of 2826 individuals with a percentage of 40.6% women and 60% men, and found significant differences, revealing a greater tendency for orthorexia in women compared to men [40]. In contrast, other studies have shown higher prevalence rates in men compared to women [25, 38, 42]. In a sample of 878 medical students, Fidan et al. [25] found a greater tendency towards ON behavior, in males, using the Turkish validation of ORTO-15. Interestingly, this study had a gender distribution similar to the study by Dell’Osso [40]. Moreover, other studies failed to find significant differences between men and women [43, 44]. The studies by Bosi et al. [43] and that of Fidan et al. [25] were performed on a population of Turkish medical students with a percentage of men and women similar to those of the previously mentioned studies; however, the sample was much smaller (*n* = 327). This disparity of results shows that gender should not be a discriminating factor when screening for ON behavior. The mixed results of the studies suggest either that gender is not a factor in orthorexia development or that it is a factor in certain sub-populations and no others, possibly related to that sub-culture’s behavior toward food [11].

Although the present study provides new, important information on the validation of the ORTO-11-ES, it is not exempt from limitations. First, the measures used consist of self-report questionnaires. Second, the population consisted of University students and, therefore, these results cannot be extrapolated beyond similar populations. Future studies should research whether the factor structures are verified in clinical samples and in other languages and cultural groups. Additionally, this tool comprises a small number of elements per factor (i.e., from three to four), and currently, it is unknown whether this is enough to cover the content of each area. This problem should be clearly addressed in future research studies evaluating the ORTO-11-ES.

Considering the limitations of the current study, we can conclude that the ORTO-11-ES may become more reliable from a statistical point of view if additional questions are added. This study offers valuable implications for future studies and clinical practice. It would be interesting to design further studies on this subject in other populations.

## Electronic supplementary material

Below is the link to the electronic supplementary material.

Supplementary material 1 (XLSX 73 KB)
